# Multiple Chronic Condition Emergency Department Visits Among U.S. Adults: Disparities at the Intersection of Intellectual and Developmental Disabilities Status and Race or Ethnicity

**DOI:** 10.1089/heq.2023.0228

**Published:** 2024-03-21

**Authors:** Hussaini Zandam, Ilhom Akobirshoev, Monika Mitra

**Affiliations:** The Lurie Institute for Disability Policy, The Heller School for Social Policy and Management, Brandeis University, Waltham, Massachusetts, USA.

**Keywords:** intellectual and developmental disabilities, race/ethnicity, multiple chronic conditions, emergency department

## Abstract

**Background:**

The study aims to examine the risk of multiple chronic condition (MCC)-related emergency department (ED) visits, MCC-related hospitalization following the ED visit, and mortality after MCC-related ED visits among adults with intellectual and developmental disabilities (IDD), adults compared with nondisabled adults by race and ethnicity, using the 2020 Healthcare Cost and Utilization Project Nationwide Emergency Department Sample.

**Methods:**

We identified IDD adults using ICD-10-CM codes, extracting 296,394 nondisabled adults and 99,538 IDD adults, of which 67,771 are white, 19,164 are black, 10,667 are Latinx, and 1936 are other race or multiple race. The control group of nondisabled adults was age-matched and sex-matched in a 1:3 case–control ratio. We conducted multilevel Poisson regression models for the binary-dependent variables and adjusted for covariates, including sociodemographic and hospital characteristics.

**Results:**

The results show that across all racial/ethnic groups, individuals with IDD have significantly higher rates of MCC-related ED visits, hospitalizations, and deaths compared with nondisabled. Moreover, the disparities are more pronounced for individuals from racial/ethnic minority groups. Black and Latinx individuals with IDD have significantly higher rates of MCC-related ED visits and poor outcomes than their white counterparts with and without IDD.

**Conclusions:**

The findings from this study highlight significant racial and ethnic disparities in the risk of MCC-related ED visits, hospitalization following the ED visit, and mortality through the ED among IDD adults. This underscores the importance of adopting a multifaceted approach that addresses the social determinants of health, enhances access to health care, improves quality of care, and enhances care coordination.

## Introduction

An emerging body of research has examined the disparities in morbidity and mortality associated with chronic conditions among people with IDD.^1,2^ Studies have found a significant gap in the average age of death between people with intellectual and developmental disabilities (IDD) and the general population.^3,4^ Other studies attributed the causes of early mortality among people with IDD to chronic diseases, including cardiovascular diseases, respiratory diseases, and cancer.^5^ Similarly, a study examining mortality among people with IDD over a 10-year period showed that early deaths in the population were associated with cardiovascular, respiratory, and neoplastic diseases.^6^ Other conditions associated with a high risk of death among people with IDD include diabetes and pneumonitis.^7^

While a U.S. study found the impact of comorbidity of chronic conditions among people with IDD,^8^ there remains a gap in research on multiple chronic conditions (MCCs) among people with IDD in the United States. A study in The Netherlands found that about 47% of older adults with IDD experienced four or more chronic conditions.^9^ Similarly, a study in Scotland found ∼28 chronic conditions and a mean number of 11.04 conditions among people with IDD.^10^ Moreover, the pattern of MCCs seen among people with IDD differs from that in the general population and spans across their entire adult life course.^10^ In Ireland, the prevalence of MCCs among adults with IDD over 40 years was found to be 71.2%, higher than the 58.6% reported for the general population older than 65 years.^11^

People with IDD from racial and ethnic minority backgrounds are disproportionately at risk of developing MCCs due to well-documented inequities, including social risks and a lack of resources necessary to navigate the health system.^12^ Rates of MCCs in the United States have been increasing across all racial/ethnic groups, with a notable racial disparity persisting from 1999 to 2018.^13^ Specifically, MCC rates are higher among black people compared with the Asian, Latino/Hispanic, and white people of the same age range. Moreover, these differences begin to emerge at 35 years and peak at 60 years.^13^ Non-Hispanic black adults in their middle years are more likely to develop MCCs at a younger age than their non-Hispanic white peers.^14^ These findings suggest a potential disparity of MCCs and poor health outcomes among people with IDD from racial and ethnic minorities.

To gain a deeper understanding of the impact of MCC-related ED visits, this study aimed to examine MCC-related emergency department (ED) visits among people with IDD from racial and ethnic backgrounds. This includes outcomes related to the following: (a) rates of MCC-related ED visits, (b) hospitalization following MCC-related ED visits, and (c) and mortality during MCC-related ED visits.

## Methods

### Data

We conducted a retrospective cohort study utilizing the 2020 Nationwide Emergency Department Sample (NEDS) from the Healthcare Cost and Utilization Project (HCUP). The HCUP-NEDS is the largest comprehensive database for ED visits across all payers in the United States. It comprises data from around 33 million ED visits annually, providing national estimates based on a sample representing ∼20% of all U.S. ED visits. The NEDS systematic sample is structured as a self-weighted sample design, similar to simple random sampling. It ensures representation across various hospital and patient factors, including urban–rural hospital locations and teaching status.^15^

### Study population

A diagnosis of IDD using the validated *International Classification of Diseases, 10^th^ Revision, Clinical Modification* (ICD-10-CM) (Supplementary Table S1) was used to identify ED visit discharge records for individuals with IDD. We restricted our sample to people aged 18–64 years. This yielded a total of 99,538 ED visit records for IDD adults, including 67,771 to non-Latinx white (hereinafter white), 19,164 to non-Latinx Black (hereinafter black), 10,667 to Latinx, 1936 to adults from other race or multiple races. We excluded observations that had diagnoses of vision,^16^ hearing,^17^ and physical^18^ disability that did not overlap with observations with IDD. The control group with no record of IDD and other types of disabilities excluded from the IDD sample (*n*=296,394) was age- and sex-matched, similar to previous research^19^ in a 1:3 case–control ratio using the greedy matching algorithm.^20^

### Measures

#### Independent variables

To categorize adults as having IDD, we examined their ED visit discharge records for the presence of IDD-related diagnosis codes (yes/no). Race/ethnicity was categorized into four groups: white, black, Latinx, and other. In addition, we created a new variable combining IDD status and race/ethnicity. This variable included the following eight categories, also representing the study cohorts: nondisabled white (referent group), white IDD (cohort 1), non-disabled black (cohort 2), black IDD (cohort 3), nondisabled Latinx (cohort 4), Latinx IDD (cohort 5), nondisabled other (cohort 6), and mixed/other IDD (cohort 7). The comparison group of nondisabled white, black, Latinx, and other adults excluded those with any disabilities, including vision, hearing, physical disabilities, and IDD.

#### Outcome variables

MCCs are defined according to the method set by the Agency for Healthcare Research and Quality (AHRQ).^21^ Specifically, MCCs refer to the co-occurrence of 2 or more 20 chronic conditions within the same individual. An ED visit is considered related to MCCs if it involves any combination of the 20 conditions in Supplementary Table S2. Chronic conditions are identified using the ICD-10-CM codes. The outcome variables included MCC-related ED visits, hospitalization following the MCC-related ED visits, and mortality during MCC-related ED visits.

#### Covariates

The model covariates consisted of sociodemographic characteristics, including age, type of health insurance (Medicare, Medicaid, private, and uninsured), and median household income for patients' zip codes ($1–$38,999, $39,000–$47,999, $48,000–$62,999, and ≥$63,000). Hospital characteristics included location and teaching status (metropolitan teaching, metropolitan nonteaching, and nonmetropolitan hospital) and hospital region (Northeast, Midwest, South, and West).

### Statistical analysis

Demographic, socioeconomic, and hospital characteristics were compared between IDD and matched nondisabled control groups in each racial and ethnic group. Differences across categorical variables and continuous variables between the two groups were evaluated using chi-squared tests and t-tests, respectively. The rate of MCC-related ED visits, hospitalizations, and mortality by IDD and race/ethnicity was reported as per 100,000 ED visits with 95% confidence intervals (CI). We applied hospital discharge weights provided by the AHRQ to estimate national rates.

We conducted a modified Poisson regression analysis to estimate the unadjusted and adjusted risk ratios (with 95% CI) for MCC-related ED visits, hospitalization following MCC-related ED visits, and mortality during MCC-related ED visits across IDD and race/ethnicity cohorts. In all analyses, we accounted for the clustering of ED visits (level 1) and within hospitals (level 2) using a multilevel modeling approach. Multilevel Poisson regression models adjusted for the patient's age, sex, insurance payer, median household income for the patient's zip code, hospital teaching status, and hospital region. We used Stata, version 17 MP (StataCorp, 2015), and a *p*-value of <0.05 as the accepted significance level for all analyses.

### Ethics approval

This study has been granted an exemption from requiring ethical review by Brandeis University's Institutional Review Board (23073R-E). The exemption is based on the fact that the research exclusively involves the use of deidentified data.

## Result

Table 1 presents the sociodemographic and hospital characteristics of patients with IDD and nondisabled stratified by race and ethnicity. In each racial and ethnic group, there were significant differences in insurance payer type, median household income for the patient's ZIP code, hospital region, and hospital teaching status between nondisabled and adults with IDD.

**Table 1.** Sample Characteristics of 18–64-Year Olds with Intellectual and Developmental Disabilities and Without Disability Stratified by Race/Ethnicity (Weighted Percentages, Nationwide Emergency Department Sample, 2020)

	White		Black		Latinx		Mixed/others	
	Disability		Disability		Disability		Disability	
	No	IDD	No	IDD	No	IDD	No	IDD
	***n*** =161,672	***n*** =67,771	***n*** =74,674	***n*** =19,164	***n*** =53,061	***n*** =10,667	***n*** =7086	***n*** =1936
Age (*p*)		**<0.006**		**0.001**		**<0.001**		**<0.001**
18–24	20.9	20.8	24.2	23.8	26.9	32.2	19.0	26.6
25–35	22.6	23.1	27.8	28.6	27.0	28.1	25.0	27.3
36–49	16.5	16.5	16.4	17.1	17.3	16.9	18.1	18.1
50–64	40.0	39.5	31.2	30.4	28.7	22.8	37.4	27.2
Sex (*p*)		**0.004**		**<0.001**		**<0.001**		**<0.001**
Male	60.8	60.9	59.4	61.8	61.5	61.7	60.9	59.1
Female	39.2	39.1	40.6	38.2	38.5	38.3	39.1	39.9
Insurance (*p*)		**<0.001**		**<0.001**		**<0.001**		**<0.001**
Medicare	11.1	50.3	10.0	37.6	5.5	29.3	5.4	33.9
Medicaid	25.8	30.3	35.8	48.0	37.7	52.4	29.4	45.6
Private	41.2	15.0	27.0	9.6	27.7	11.3	50.0	17.1
Uninsured	21.9	4.3	27.4	4.8	29.1	6.9	15.1	3.4
Household income (*p*)		**<0.001**		**<0.001**		**<0.001**		**<0.001**
$1–$38,999	31.4	29.8	53.5	47.4	36.3	36.4	14.2	11.4
$39,000–$47,999	29.2	28.9	23.4	24.1	28.5	27.1	17.9	19.5
$48,000–$62,999	21.5	22.4	14.1	16.8	22.0	23.2	24.5	24.1
63,000+	17.9	18.9	9.0	11.7	12.9	13.4	43.4	45.1
Region of hospital (*p*)		**<0.001**		**<0.001**		**<0.001**		**<0.001**
Northeast	15.6	23.2	12.9	19.5	17.0	22.5	16.0	17.2
Midwest	26.2	28.9	21.8	24.8	6.6	6.4	11.7	12.2
South	40.9	33.0	55.1	46.6	33.5	30.4	12.8	9.5
West	17.2	14.8	10.1	9.1	42.8	40.8	59.5	61.0
Teaching status of hospital (*p*)		**<0.001**		**<0.001**		**<0.001**		**<0.001**
Metropolitan nonteaching	25.5	21.6	19.9	16.2	24.3	21.0	19.4	16.9
Metropolitan teaching	54.6	62.0	70.8	75.7	71.1	75.3	76.4	79.3
Nonmetropolitan hospital	19.7	16.4	9.4	8.1	4.6	3.7	4.2	3.8

Source: Healthcare Cost and Utilization Project, National Emergency Department Sample (HCUP-NEDS) data, 2020.

Boldface indicates statistical significance (*p*<0.05).

IDD, intellectual and developmental disabilities.

### Differences in MCC-related ED visit outcomes between adults with no disabilities and IDD within racial and ethnic groups

In every racial and ethnic group, individuals with IDD had higher rates of MCC-related ED visits, hospitalizations, and mortality compared with nondisabled cohorts (Table 2).

**Table 2.** Rates of Multiple Chronic Condition-Related Emergency Department Visits, Hospitalization, and Mortality by Race/Ethnicity and Disability Status

Cohorts	ED visits (weighted)	***p***	Hospitalization (weighted)	***p***	Mortality (weighted)	***p***
	Per 100,000 (CI)		Per 100,000 (CI)		Per 100,000 (CI)	
White		<0.001		<0.001		<0.001
Non-IDD	36,502^***^ (13,030–19,310)		11,042^***^ (9081–12,331)		281^***^ (267–293)	
IDD	50,330 (31,244–35,983)		25,221 (22,504–26,927)		785 (771–794)	
Black		<0.001		<0.001		<0.001
Non-IDD	46,810^***^ (45,610–48,380)		14,062^***^ (13,810–15,322)		370^***^ (362–388)	
IDD	52,040 (50,312–54,089)		30,410 (28,070–34,760)		985 (965–993)	
Latinx		<0.001		<0.002		<0.001
Non-IDD	35,821^***^ (34,504–36,330)		15,060^**^ (14,504–16,627)		290^***^ (258–303)	
IDD	49,370 (46,049–51,704)		27,001 (27,729–30,785)		857 (844–878)	
Other/mixed race		<0.010		<0.010		<0.001
Non-IDD	39,584^**^ (37,981–42,451)		9860^**^ (8751–10,115)		311^***^ (301–327)	
IDD	48,320 (45,040–49,981)		25,083 (22,814–26,891)		830 (819–865)	

*N*=395,932. Source: Health Care Cost and Utilization Project National Emergency Department Sample (HCUP-NEDS) data, 2020.

^***^ *p*<0.001, ^**^p<0.01, ^*^p<0.05.

ED, emergency department.

### IDD status and risk for MCC-related ED visits

In unadjusted analyses, compared with non-IDD whites, white individuals with IDD were at a higher risk for MCC-related ED visits (RR=1.78, 95% CI: 1.75–1.81), hospitalization following ED visits (RR=2.71, 95% CI: 2.65–2.87), and mortality during ED visit (RR=3.78, 95% CI: 3.67–4.11). The risks remained robust and statistically significant even after adjusting for sociodemographic and hospital characteristics.

### Race and ethnicity and risk for MCC-related ED visits

Compared with non-IDD white, black and Latinx nondisabled were at a significantly higher risk for MCC-related ED visits, hospitalization following ED visits, and mortality (Table 3). However, nondisabled mixed/other race individuals were at a greater risk for mortality from MCC-related ED hospitalization compared with white nondisabled.

**Table 3.** Unadjusted and Adjusted Rate Ratios for Multiple Chronic Condition-Related Emergency Department Visits, Hospitalizations, and Mortality by Race/Ethnicity and Disability Status

Cohorts	ED visits		Hospitalizations		Mortality	
	**Unadjusted**	**Adjusted**	**Unadjusted**	**Adjusted**	**Unadjusted**	**Adjusted**
White nondisability	Referent	Referent	Referent	Referent	Referent	Referent
White IDD	1.78^***^ 1.75–1.81	1.55^***^ 1.40–2.07	2.71^***^ 2.65–2.87	2.55^***^ 2.51–2.62	3.78^***^ 3,67–4.11	3.52^***^ 3.45–3.61
Black nondisability	1.52^***^ 1.41–1.65	1.28^***^ 1.21–1.75	1.76^**^ 1.69–1.92	1.61^***^ 1.47–1.66	2.09^***^ 1.87–2.14	1.82^***^ 1.74–1.95
Black IDD	2.31^***^ 2.25–2.57	2.11^***^ 1.97–2.24	3.12^***^ 3.09–3.18	2.88^***^ 2.80–2.96	4.21^***^ 4.12–4.90	4.02^***^ 3.88–4.30
Latinx nondisability	1.67^*^ 1.61–1.73	1.55^**^ 1.37–1.56	2.23^**^ 2.18–2.29	1.20^**^ 1.19–1.24	0.91 0.78–1.91	1.02 0.91–1.21
Latinx IDD	2.55^***^ 2.49–2.62	2.27^***^ 2.13–2.36	3.03^***^ 2.90–3.37	2.51^***^ 2.48–2.60	4.06^***^ 3.75–4.33	3.82^***^ 3.71–3.99
Others/mixed non-IDD	1.13 1.04–1.30	0.92 0.77–0.98	1.19^*^ 0.82–1.76	1.02 0.61–1.33	1.31^**^ 1.27–1.42	1.25^*^ 1.09–1.33
Others/mixed IDD	1.74^***^ 1.69–1.80	1.66^***^ 1.50–1.83	2.52^***^ 2.41–2.88	2.33^***^ 2.24–2.67	3.81^***^ 3.80–3.94	3.77^***^ 3.58–3.94

*N*=395,932. Non-IDD whites were the reference group. Multivariate analysis adjusted for age, sex, insurance payer, median household income for the patient's zip code, and hospital region. Source: Health Care Cost and Utilization Project National Emergency Department Sample (HCUP-NEDS) data, 2020.

^***^ *p*<0.001, ^**^p<0.01, ^*^p<0.05.

### Risk for MCC-related ED visits at the intersection of race/ethnicity and IDD status

The combined risk of IDD and racial/ethnic minority status on the MCC-related ED visits is additive specifically among black IDD (RR=2.31) and Latinx IDD (RR=2.55) cohorts, indicating that their risk is higher than that of white individuals with IDD (RR=1.78) or individuals who are black (RR=1.52) and Latinx (RR=1.67) alone. This pattern of additive effects is observed across all minority groups in MCC-related hospitalizations and deaths.

## Discussion

This study highlights significant disparities in the risk of MCC-related ED visits, hospitalizations following ED visits, and deaths during ED visits among racial and ethnic minorities with IDD. Irrespective of their racial/ethnic group, individuals with IDD experience significantly higher rates of MCC-related ED visits, hospitalizations, and deaths compared with the nondisabled comparison group. Moreover, the disparities are more pronounced for individuals from racial/ethnic minority groups. For instance, black and Latinx individuals with IDD face significantly higher rates of MCC-related hospitalizations and death than their white counterparts with IDD.

A higher MCC-related ED utilization rate may indicate poor care management and inadequate access to care. Efficient care management is crucial in ensuring patients receive well-coordinated and appropriate primary care to prevent unnecessary ED visits.^22,23^ This disparity in our findings could be attributed to various factors. The disparity we observed between IDD and race/ethnicity is likely because of the higher prevalence of MCCs among people with IDD and racial and ethnic minorities. Earlier studies have shown that people with IDD are disproportionately more likely to develop MCCs.^9–11,24,25^

Racial and ethnic disparities in MCCs are well documented in the United States, with higher prevalence among the black and Latino/Hispanic populations.^13,14^ While there is a lack of information specifically on the prevalence of MCCs among people with IDD from racial and ethnic minority groups, studies have shown more significant disparities in health at the intersection of IDD and race/ethnicity.^26–28^ These disparities may contribute to the higher rate of MCC-related ED visits among individuals with IDD among racial and ethnic minority backgrounds.

The complexity of MCCs often results in individuals requiring more frequent primary and specialty care, along with more complex medication regimens. The existing disparities in access to care and systemic issues with the quality of care received by people with IDD and racial and ethnic minority groups can potentially exacerbate the risk of ED visits in this population.^29,30^ Compared with white non-IDD, those with IDD and racial and ethnic minorities are much more likely to have coverage from public health insurance (primarily Medicaid and Medicare). Previous studies have indicated that MCC complications are more prevalent and severe among racial minorities and adults covered by Medicare and Medicaid, which constitutes a substantial part of our sample.^31–33^

Publicly funded health insurance often has limited provider options, leading to difficulty accessing specialized providers or longer appointment wait times. Furthermore, issues with medications such as drug–drug and disease interactions, therapeutic duplication, and polypharmacy associated with MCCs could potentially disproportionately affect people with IDD from racial/ethnic minority backgrounds.^34^

There are other well-documented biases and inequities at the point of care that may result in the disparities experienced by people with IDD, especially marginalized racial and ethnic groups. These individuals may face prejudices linked to both race/ethnicity and disability. Adults with disabilities who are members of underserved racial and ethnic groups are likely to face physical and attitudinal barriers to quality care from their disability and race/ethnicity.^35^ Issues such as language barriers have been shown to substantially complicate the coordination of care for migrant IDD family members with complex health care needs.^36^ Poor quality of communications with health care professionals has been reported by parents of children with IDD who are African American/black or Hispanic/Latino than white parents.^37^

These issues highlight the urgent need to address and rectify the systemic biases and barriers that people with IDD and racial and ethnic minority backgrounds encounter in the health care system. By promoting cultural competence, improving communication, and addressing attitudinal barriers, we can create a more inclusive and equitable health care environment for all individuals regardless of race, ethnicity, or disability status.

Our findings underscore the need to address the health disparities of people with IDD, especially those from marginalized racial and ethnic groups who may encounter biases related to both race/ethnicity and disability. It is crucial to direct attention toward understanding how disability-related disparities interact with those associated with race/ethnicity and other dimensions of inequity to guide research, policy, and interventions.

### Limitations

The study comes with some limitations that warrant consideration. It is possible that some individuals with IDD who visited the ED were not given an ICD-10 IDD diagnosis, as the primary focus of their visit to the ED was unrelated to their IDD. Thus, these claims only include people who received a diagnosis of IDD during their ED visit, potentially excluding those with IDD who were not diagnosed. The unit of study was based on ED visits rather than the individual. This means a person could be represented multiple times in the data if they were in the ED more than once a year. Our measure of socioeconomic status, which relies on household income data from a patient's five-digit ZIP code, may introduce inaccuracies.

Our list of MCCs includes conditions such as depression and substance abuse, which may also be classified as acute. This reflects the complex nature of these conditions, which can present both acute episodes and chronic management challenges. Furthermore, the inclusion of certain psychiatric conditions and the exclusion of others, such as schizophrenia and bipolar disorder, point to the need for a more nuanced approach to categorizing chronicity and understanding its implications for emergency care. The heterogeneity within the MCC group is significant, especially when considering individuals with IDD, who may present with what is sometimes referred to as a dual diagnosis. This heterogeneity extends to the reasons for ED visits, the unique challenges faced in emergency settings, and the spectrum of treatment needs

Lastly, we also consider the potential variability in the recording of IDD and other conditions by race. This likelihood introduces an element of inconsistency that might affect our study's findings. We could not determine the severity of MCC-related ED visits, potentially limiting the strength of our findings. Despite these limitations, to our knowledge, this is the first study investigating MCC-related ED visits among adults with IDD using extensive, nationally representative ED visit discharge records.

## Conclusion

The findings from this study reveal notable racial and ethnic disparities in the risk of MCC-related ED visits, hospitalization following the ED visit, and death during the ED visit among adults with IDD. Their heightened risk of poor outcomes from MCCs among IDD from racial and ethnic minorities underscores the need for a comprehensive approach that focuses on social determinants of health, enhancing access to health care and improving the quality and coordination of health care. To effectively mitigate these disparities, it is critical to implement multifaced strategies that tackle the underlying systemic issues contributing to these inequities.

## Supplementary Material

Supplemental data

Supplemental data

## Abbreviations Used

AHRQ: Agency for Healthcare Research and Quality
CI: confidence intervals
ED: emergency department
HCUP: Healthcare Cost and Utilization Project
IDD: intellectual and developmental disabilities
MCCs: multiple chronic conditions
NEDS: Nationwide Emergency Department Sample
