# Supplementary material for: Multiple Chronic Condition Emergency Department Visits Among U.S. Adults: Disparities at the Intersection of Intellectual and Developmental Disabilities Status and Race or Ethnicity
Source: Health Equity. 2024 Mar 21;8(1):198–205. doi: 10.1089/heq.2023.0228 (PMC10979663; doi:10.1089/heq.2023.0228)
Supplement: Supplemental data [file Suppl_TableS1.docx]

**Supplemental Table 1. Intellectual and Developmental Disabilities Subgroup Definitions and Corresponding International Classification of Diseases, Tenth Revision, Clinical Modification Codes**

| Description | ICD-10 |
| --- | --- |
|  |  |
| Mild Mental Retardation | F70 |
| Moderate Mental Retardation | F71 |
| Severe Mental Retardation | F72 |
| Profound Mental Retardation | F73 |
| Unspecified Mental Retardation | F79 |
| Fragile X Syndrome | Q99.2 |
| Prader-Willi Syndrome | Q87.1 |
| Down Syndrome | Q90.9 |
| Rett Syndrome | F84.2 |
|  | G31.81 |
| G31.81 |  |
| Lesch Nyhan | E79.1 |
|  | E79.8 |
| Cri du Chat | Q93.4 |
| Autistic Disorder | F84.0 |
|  |  |
| Childhood Disintegrative Disorder | F84.3 |
| Other Specified PDDs | F84.5 |
| Unspecified PDD | F84.9 |
| Tuberous Sclerosis | Q85.1 |
| Fetal Alcohol Syndrome | P04.3 |
|  | Q86.0 |
| CP Athetoid | G80.3 |
| CP Diplegic | G80.1 |
| CP Hemiplegic | G80.2 |
| CP Quadriplegic | G80.0 |
| CP Monoplegic | G80.8 |
| Other CP | G80.2 |
| Infantile CP | G80.8 |
| CP Spastic | G80.9 |
| CP Spastic Non-Congenital | G83.81 |
|  | G83.84  G83.89 |
