# Supplementary material for: Multiple Chronic Condition Emergency Department Visits Among U.S. Adults: Disparities at the Intersection of Intellectual and Developmental Disabilities Status and Race or Ethnicity
Source: Health Equity. 2024 Mar 21;8(1):198–205. doi: 10.1089/heq.2023.0228 (PMC10979663; doi:10.1089/heq.2023.0228)
Supplement: Supplemental data [file Suppl_TableS2.docx]

**Supplemental Table 2. Chronic Conditions Definitions and Corresponding International Classification of Diseases, Tenth Revision, Clinical Modification Codes**

| Description | ICD-10 |
| --- | --- |
| Cancer | Colorectal cancer: C17-C18, C21, Z85. 038 |
|  | Uterine cancer: C54, C55, D07.0, D25, Z85.42. |
|  | Pancreatic: C25, D13, Z85.07 |
|  | Stomach cancer: C16, D13.1, Z85.020, Z85.028 |
|  | Genitourinary tract cancer: C61, C64-C68, Z85.520, Z85.528, Z85.53, Z85.54, Z85.51, Z85.59 |
|  | Ovarian cancer: C56-C57, C48, Z85.43 |
|  | Skin cancer : C34, C43, Z85.820, Z85.828 |
|  | Endocrine cancer: C73, C74, C75, Z85.850, Z85.858  Bone and marrow cancer: C40, C41,C71, Z85.830, |
|  | Other cancer: C69, C70, C71, Z85.840, Z85.841, Z85.848 |
| Hypertension | I10 |
| Diabetes mellitus | E10, E11, E12, E13, E14. |
| Ischemic heart diseases | I20, I21, I22, I23, I24, I25. |
| Heart failure | I50 |
| Chronic liver disease | K70-K76 |
| Asthma | J45 |
| HIV/AIDS | B20-B24 |
| Chronic obstructive pulmonary disease | J44 |
| Chronic pain | G89 |
| Depression | F32, F33 |
| Obesity | E66 |
| Arthritis | MO5-MO19, M45 |
| Osteoporosis | M80, M81, M82 |
| Stroke | I60-I69 |
| Chronic Kidney Disease | N18 |
| Dementia | FO0-FO3 |
| Alzheimer's Disease | G30 |
| Parkinson’s Disease | G20, G21 |
| Alcohol and substance abuse | F10-F19 |
|  |  |
